# Preface: Development and evaluation of improved strains of insect pests for sterile insect technique (SIT) applications

**DOI:** 10.1186/1471-2156-15-S2-I1

**Published:** 2014-12-01

**Authors:** Kostas Bourtzis, Jorge Hendrichs

**Affiliations:** 1 Insect Pest Control Laboratory, Joint FAO/IAEA Programme of Nuclear Techniques in Food and Agriculture, P.O. Box 100, 1400 Vienna, Austria; 2 Insect Pest Control Section, Joint FAO/IAEA Division of Nuclear Techniques in Food and Agriculture, P.O. Box 100, 1400 Vienna, Austria

## 

Pest insects represent an enormous burden to agricultural, veterinary and human health activities in every continent. Their sustainable management is of the utmost importance for the effective utilization of the increasingly limited agricultural resources and for the well-being of humankind. In response to requests from Member States, the Joint FAO/IAEA Insect Pest Control Subprogramme aims to develop, validate and transfer to its Member States environment-friendly technologies to control populations of insect pests and disease vectors including the sterile insect technique (SIT) as a component of area-wide integrated pest management (AW-IPM) programmes.

The SIT has the ability to suppress insect pest populations, or to prevent, contain or even eradicate the establishment of new outbreaks of invasive pests. The operational use of the SIT in AW-IPM programmes continues to increase and there are currently several active programmes in almost all continents against agricultural pests, mainly fruit flies and lepidopteran (moth) pests, but also other agricultural pests or disease vectors of livestock and humans. However, managers have indicated that programme efficiency can still be considerably enhanced when certain components of the technology are improved such as, for example, the availability of genetic sexing strains (GSS) against new target species or the development of strains carrying genetic markers which would allow the differentiation of released sterile insects from wild populations.

To address these gaps, the Joint FAO/IAEA Insect Pest Control Subprogramme initiated the Coordinated Research Project (CRP) "Development and Evaluation of Improved Strains of Insect Pest for SIT applications". The activities of this CRP were focused on four main areas: (1) insect genetics and transformation, (2) sex determination, (3) development of novel sexing systems and (4) improvement and evaluation of existing strains for SIT applications. This special issue presents part of the progress accomplished in the frame of this CRP.

Using classical genetic approaches, CRP participants succeeded in developing and characterizing new GSS against major agricultural pests such as the Mexican fruit fly (mexfly) *Anastrepha ludens *[1] and the carambola fly, *Bactrocera carambolae *[2]. Another major achievement was the development and evaluation of male-only strains of the Australian sheep blowfly Australian sheep blowfly *Lucilia cuprina *using modern biotechnology approaches [3].

Using similar molecular genetic approaches, male-specific Y-linked markers were developed which could enhance biologically-based control of the mexfly [4]. In addition, Y-linked, male-biased genes and male-specific phosphorylated proteins were characterized in the Mediteranean fruit fly (medfly) *Ceratitis capitata *which opens the way for new markers as well as the elucidation of sex determination pathways [5,6]. Transcriptomic approaches were also employed to elucidate these pathways in two other important species, *Bactrocera jarvisi *and the olive fruit fly *Bactrocera oleae *[7,8].

This supplement also includes a very thorough review about the currently available genetic, molecular and microbial tools for improved SIT applications against Australian endemic pest tephritids [9] as well as two additional contributions on the medly *C. capitata*: the first focuses on polyandry phenomena, a critical factor for the implementation of the SIT, while the second reviews recent advances on functional genomics and how they might contribute on pest control [10,11].

There is an urgent need to develop and apply the SIT against new target agricultural pest species such as the South American fruit fly *Anastrepha fraterculus*. This requires a very good knowledge of the biology and the genetics of the species. Progress in this area is herein summarized in a review article [12]. In addition, two original research articles describe the development of microsatellite markers and their use to monitor changes which might be happening upon the laboratory colonization of an *A. fraterculus *population for mass rearing and SIT applications [13,14].

Cytogenetics analyses can play a significant role in many aspects of basic and applied research including mapping a single gene in chromosomes to resolving species boundaries within species complexes and this special issue presents such examples in two manuscripts: the first presents a TSA-FISH approach which was developed to map single-copy genes in the codling moth *Cydia pomonella *while the second describes how comparative cytogenetic analysis can support SIT applications by contributing to species delimitation in the *Bactrocera dorsalis *species complex [15,16].

During the last years, new strains based on biotechnological engineering have been developed for SIT or related applications. These transgenic strains carry a single killing system and major concerns have been raised with regards to resistance development. The last manuscript of this special issue presents a perspective about how "redundant killing", through an additional independent conditional expression system, could be developed and used to biotechnologically enhance SIT strategies [17].

In conclusion, significant progress was achieved in the frame of the CRP on "Development and Evaluation of Improved Strains of Insect Pest for SIT Applications" as shown in the manuscripts included in this special issue. We expect that the articles presented here will be a source of inspiration and a stimulus to the scientific community for additional basic and, particularly, applied research in this field.

Last but not least, the Editors of this special issue would like to acknowledge the outstanding scientific contributions of Dr. Gerald Franz in this field. Dr. Franz was the leader of the Genetics Group at the Joint FAO/IAEA Insect Pest Control Laboratory for close to twenty-five years, where he played a critical role in the initiation in 2009 of this CRP and in coordinating all its activities until his retirement in September 2012. Most importantly, using classical genetic approaches, Dr. Franz and his collaborators exploited mutations such as *white pupal color *and *temperature sensitive lethal *to pioneer the development of several generations of medfly genetic sexing strains which allow the elimination of females at the embryonic stage at an industrial level and with a very high accuracy. In response to being able to mass-produce only sterile males and of significantly increasing their effectiveness in the absence of sterile females, all SIT programmes in the world against this pest have been using since the 1990s these GSS, namely VIENNA 7 and VIENNA 8, in their mass-rearing factories, resulting in the production and the release of billions of sterile males on a weekly basis. This has represented major cost savings and benefits of increased SIT effectiveness for programmes in developing and developed Member States. This catalytic achievement has significantly improved the operational SIT programmes, particularly as concerns their effectiveness and cost efficiency, in developing and developed FAO and IAEA Member States. In an elegant study, which included deletion mapping, Dr. Franz and his group were also able to map the male determining factor in the Y chromosome of *C. capitata*, a major achievement which now waits for the next step: the isolation of the Maleness factor [18].

During the implementation of this CRP, an eminent member of our consortium, Dr. Javaregowda Nagaraju, a renowned scientist who spent his lifetime researching silkworms, sadly passed away on the very last day of the year 2012. He was a researcher with an endless challenging spirit, a great teacher and a most beloved friend, due to his very warm, enthusiastic and welcoming nature. His studies on the silkworm had such impact, that his laboratory became one of the leading centres in this field in India and in the world. His research appeared in over 100 papers and reviews in high quality journals. This special issue is dedicated to his memory.

## Competing interests

The authors declare that they have no competing interests.

## Notes

This article has been published as part of *BMC Genetics *Volume 15 Supplement 2, 2014: Development and evaluation of improved strains of insect pests for SIT. The full contents of the supplement are available online at http://www.biomedcentral.com/bmcgenet/supplements/15/S2.

Publication of this supplement was funded by the International Atomic Energy Agency. The peer review process for articles published in this supplement was overseen by the Supplement Editors in accordance with BioMed Central's peer review guidelines for supplements. The Supplement Editors declare that they have no competing interests.
